# Lippia origanoides essential oil possesses anticonvulsant effect in pentylenetetrazol-induced seizures in rats: a behavioral, electroencephalographic, and electromyographic study

**DOI:** 10.3389/fphar.2023.1289336

**Published:** 2023-11-28

**Authors:** Daniella Bastos de Araújo, Anthony Lucas Gurgel do Amaral, Suzane Maia da Fonseca, Keyla Rodrigues de Souza, Allane Patrícia Santos da Paz, Vanessa Jóia de Mello, Gabriela Brito Barbosa, Maria Klara Otake Hamoy, Moisés Hamoy

**Affiliations:** Laboratory of Pharmacology and Toxicology of Natural Products, Institute of Biological Science, Federal University of Pará, Belém, Brazil

**Keywords:** seizures, pentylenetetrazol, electrocorticographic recordings, essential oil, Lippia origanoides

## Abstract

Epilepsy is a neuronal disorder characterized by abnormal excitability of the brain, leading to seizures. Only around 66% of the epileptic patients respond adequately to treatment with existing conventional anticonvulsants, making it necessary to investigate new antiepileptic drugs. The growing research into natural products and their pharmacological properties has become increasingly promising, particularly in the study of essential oils, which are already widely used in popular culture for treating various diseases. The present study evaluated the anticonvulsant effects of Lippia origanoides essential oil (LOEO) (100 mg/kg i. p.) compared to diazepam (DZP) (5 mg/kg i. p.), and the combined administration of these two substances to control convulsions induced by pentylenetetrazol (PTZ) (60 mg/kg i. p.). This evaluation was carried out using 108 male Wistar rats, which were divided into two experiments. Experiment 1–Behavioral assessment: The animals were divided into 4 groups (*n* = 9): (I) saline solution + PTZ, (II) DZP + PTZ, (III) LOEO + PTZ, (IV) LOEO + DZP + PTZ. The convulsive behavior was induced 30 min after the administration of the tested anticonvulsant drugs, and the observation period lasted 30 min. Experiment 2- Electrocorticographic evaluation: The animals were divided into 8 groups (*n* = 9): (I) saline solution; (II) LOEO; (III) DZP; (IV) LOEO + DZP; (V) saline + PTZ, (VI) DZP + PTZ (VII) LOEO + PTZ, (VIII) LOEO + DZP + PTZ. PTZ was administered 30 min after LOEO and DZP treatments and electrocorticographic activity was assessed for 15 min. For the control groups, electromyographic recordings were performed in the 10th intercostal space to assess respiratory rate. The results demonstrated that Lippia origanoides essential oil increased the latency time for the appearance of isolated clonic seizures without loss of the postural reflex. The animals had a more intense decrease in respiratory rate when combined with LOEO + DZP. EEG recordings showed a reduction in firing amplitude in the LOEO-treated groups. The combining treatment with diazepam resulted in increased anticonvulsant effects. Therefore, treatment with Lippia origanoides essential oil was effective in controlling seizures, and its combination with diazepam may represent a future option for the treatment of difficult-to-control seizures.

## 1 Introduction

Epilepsy is a neurological disorder that affects around 45.9 million people globally and is characterized by a predisposition to suffer spontaneous seizures (Fisher et al., 2014). Its pathophysiology consists of the appearance of abnormal foci of cerebral electrical activity caused by the imbalance between excitatory and inhibitory neurotransmitters in the central nervous system, in such a way as to make it prone to functioning in an excessive oscillatory pattern. Conventional antiepileptic drugs act through these two pathways, either by enhancing inhibitory neurotransmitters or reducing excitatory signaling (Fisher et al., 2005; Sultana et al., 2021).

Currently available drug therapies are effective in only 66% of cases in developed countries (Duncan et al., 2006; Brodie et al., 2012) and are associated with various side effects (Perucca and Gilliam, 2012), highlighting the need for research to identify alternative treatments that target seizure mechanisms and have minimal side effects (Sultana et al., 2021).

One promising option is the use of essential oils (EOs) in the treatment of epilepsy. Essential oils are volatile substances extracted from plant parts, made up of a mixture of various components with therapeutic properties, widely used in popular culture to treat various ailments (de Almeida et al., 2011; Dobetsberger and Buchbauer, 2011). Recent studies have shown that several essential oils from aromatic plants have potential neuroprotective effects in age-related neurodegenerative diseases such as Alzheimer’s and dementia and other neurological diseases such as anxiety, depression, epilepsy and seizures (Ayaz et al., 2017; Rashed et al., 2021; Sattayakhom et al., 2023).

Behavioral assessment and electrocorticography are of paramount importance in evaluating and comparing the changes caused by neuronal discharge that trigger seizures and epilepsy. In recent studies using natural products with potential anticonvulsant activities, data have shown that in the behavioral assessment, there were increases in seizure latencies and in the seizure threshold, confirmed by electrocorticographic records, along with a decrease in the peak and energy of the waves (Souza-Monteiro et al., 2015; Hamoy et al., 2018; Nascimento et al., 2022; Muto et al., 2022).

Lippia origanoides is a shrub with a strong aroma found mainly in the Amazon territory (Pascual et al., 2001; Oliveira et al., 2014) with medicinal properties well-known in popular culture (Siqueira-Lima et al., 2019). In Central America and Colombia, it is used to treat respiratory diseases, gastrointestinal discomfort such as gastralgia, nausea and antiseptic. In the countryside of Pará, in Brazil, Lippia origanoides, known as “salva-do-marajó,” is commonly administered to combat intestinal colic, indigestion, diarrhea, burns, vaginal discharge, menstrual cramps, and fever (Oliveira et al., 2014). It is also notable for its use in food preparation, and in Venezuela, it is employed as an appetite stimulant (Morton, 1981).

Regarding the anticonvulsant properties of LOEO (Lippia origanoides essential oil), no studies have been found directly linking this plant to such effects. However, there are articles that suggest anticonvulsant effects of Lippia alba due to the high presence of flavonoids in its composition (Zétola et al., 2002; Neto et al., 2009; Siqueira-Lima et al., 2019), as well as L. Citriodora (Rashidian et al., 2016).

In this context, the objective of the present work was to evaluate the treatment with essential oil of *L. origanoides* in the control of seizures triggered by pentylenetetrazol and compare its effects with those of diazepam, through behavioral analysis, electrocorticography and assessment of respiratory movements (electromyogram).

## 2 Materials and methods

### 2.1 Animals

For the execution of this research, 108 adult Wistar rats were obtained from the Central Bioterium of the Federal University of Pará. All animals were housed under controlled conditions, with a temperature of approximately 23°C–25°C and a light-dark cycle of 12/12 h, receiving filtered water and rat food on free demand. The work was performed at the Laboratory of Pharmacology and Toxicology of Natural Products (Laboratório de Farmacologia e Toxicologia de Produtos Naturais)—ICB—UFPA. The project was approved by the Animal Ethics Committee (CEUA—UFPA) number 1395260821.

### 2.2 Drugs used

Lippia origanoides essential oil (LOEO) was purchased from Olinda pharmaceutical company (essential oils) and administered intraperitoneally at a dose of 100 mg/kg, while Diazepam (DZP) 10 mg/2 mL (União Química, Embu-Guaçu, SP, Brazil), was administered at a dose of 5 mg/kg intraperitoneally (i.p). Ketamine hydrochloride (50 mg/kg i. p.) was purchased from Köing Laboratory (Santana de Parnaíba, SP, Brazil), xylazine hydrochloride (5 mg/kg i. p) was purchased from Vallée Laboratory (Montes Claros, MG, Brazil), while the local anesthetic lidocaine was obtained from Hipolabor Laboratory (Sabará, MG, Brazil). Pentylenetetrazol (PTZ) was obtained from Sigma Chemical Co. (St. Louis, MO, United States) and administered intraperitoneally at a dose of 60 mg/kg (Santos et al., 2021; Muto et al., 2022).

### 2.3 Test to obtain the dose of LOEO used

To obtain the dose used of Lipia Origanoides extract (LOEO), a fixed time of 30 min was considered to achieve muscle relaxation and animal sedation behavior at tested doses of 50 mg/kg, 100 mg/kg, 150 mg/kg, and 200 mg/kg i. p. the best response obtained was 100 mg/kg i. p. as higher doses caused myorelaxation with manifestation of respiratory depression. Therefore, a dose of 100 mg/kg i. p. was used 30 min before the onset of the seizure using PTZ (60 mg/kg).

### 2.4 Experimental design

**FIGURE 1:**
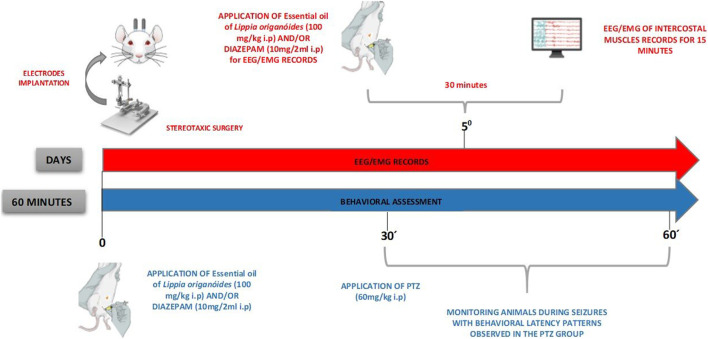
Experimental design. PTZ, pentylenetetrazol; EEG, electroencephalograph; EMG, electromyogram.

The animals were kept at the research center for at least 7 days before the experiment for adaptation and acclimatization. The electrodes were implanted in the cortex 5 days before the application of the treatments. For the behavioral assessment the animals were divided into 4 groups (*n* = 9): (I) saline + PTZ, (II) DZP + PTZ, (III) LOEO + PTZ, (IV) LOEO + DZP + PTZ. The convulsant drug PTZ was administered 30 min after administration of the drugs tested as anticonvulsants and the observation time for behavior analysis was 30 min (Figure 1).

For electrocorticographic evaluation, animals were divided into 8 groups (*n* = 9): (I) saline; (II) LOEO; (III) DZP; (IV) LOEO + DZP; (V) saline + PTZ, (VI) DZP + PTZ (VII) LOEO + PTZ, (VIII) LOEO + DZP + PTZ. PTZ was administered 30 min after treatments and electrocorticographic activity was assessed for 15 min (Figure 1).

### 2.5 Evaluation of respiratory activity during sedation

For the analysis of respiratory frequency and muscle contraction power (Santos et al., 2021), electrodes were conjugated 2 mm apart and were prepared with a length of 2 mm and a diameter of 0.2 mm. These electrodes were inserted into the 10th intercostal space to record muscle activity. The recordings were conducted for a duration of 5 min for the Control, LOEO, DZP, and LOEO/DZP groups.

### 2.6 Description of seizure related behavior

The animals’ behavior was monitored during the seizures and compared with latency patterns of behaviors observed in the PTZ-induced group (de Almeida. et al., 2020). Latency was measured concerning the initiation of the following behaviors: (I) whisker piloerection, (II) orofacial movements, (III) generalized tremor, (IV) anterior limb spasms, (V) isolated clonic seizures without loss of postural reflex, (VI) generalized clonic seizures with transient loss of postural reflex, and (VII) tonic-clonic seizures with complete loss of postural reflex.

### 2.7 Electrocorticographic recordings and data analysis

The animals were anesthetized and placed in a stereotaxic apparatus for the implantation of electrodes (with an exposed tip diameter of 1.0 mm) onto the dura mater above the prefrontal cortex at the coordinates of bregma–0.96 mm and ±1.0 mm lateral. The electrodes were secured using dental acrylic cement. Data were recorded using the electrodes with the assistance of a digital data acquisition system composed of a high-impedance amplifier (Grass Technologies, P511, United States), set with a filtering range of 0.3 Hz to 0.3 KHz. The data were monitored using an oscilloscope (Protek, Model 6510) and continuously digitized at a rate of 1 KHz by a computer equipped with a data acquisition board (National Instruments, Austin, TX).

During the recording sessions, the animals were confined within acrylic boxes (20 cm × 45 cm × 15 cm), and ECoG activity was recorded for 15 min immediately after the application of PTZ or physiological solution. The data collected through the digital data acquisition system were analyzed offline. The analyses were performed in frequencies up to 40 Hz and then divided into five bands: delta (1–4 Hz), theta (4–8 Hz), alpha (8–12 Hz), beta (12 Hz–28 Hz), and gamma (28 Hz–40 Hz) [30].

The characterization of the aspects of neuronal hyperexcitability in seizures caused by PTZ, as well as the reversal of the condition by the control drugs, were performed using the Signal ^®^ 3.0 and Pyton 5.0 programs, which allowed the analysis of the frequency domain of brain waves, in addition to the visual inspection of wave patterns.

### 2.8 Chromatographic analysis of Lippia origanoides essential oil

Gas Chromatography-Mass Spectrometry (GC-MS) analysis, with Agilent Model MSD 5977B apparatus, was carried out by the company Olinda (essential oils) to certify the chromatographic analysis of *Lippia origanoides*. The analysis was performed on a batch with manufacturing date of February 2022, labeled as lot: 180002.

Organoleptic Properties: The essential oil appeared as a liquid with a golden-yellow color, free of impurities. It exhibited a pungent, fresh, and herbal scent, and had a density of 0.935 at 20°C. It originated from Brazil and was obtained through steam distillation.

The components were identified based on the Chemical Abstracts Service (CAS) registry number, which assigns a unique number to each chemical compound described in literature. The major components identified were Thymol (57.46%) and Carvacrol (1.42%).

### 2.9 Statistical analysis

The results were subjected to descriptive statistics, including mean and standard deviation. One-way Analysis of Variance (ANOVA) was employed, followed by Tukey’s *post hoc* test. A significance level of *p < 0.05, **p < 0.001, and ***p < 0.0001 was adopted. The analyses were performed using GraphPad Prism, version 9 (GraphPad Software Inc., San Diego, CA, United States).

## 3 Results

### 3.1 Respiratory evaluation after administration of isolated and associated drugs

**FIGURE 2:**
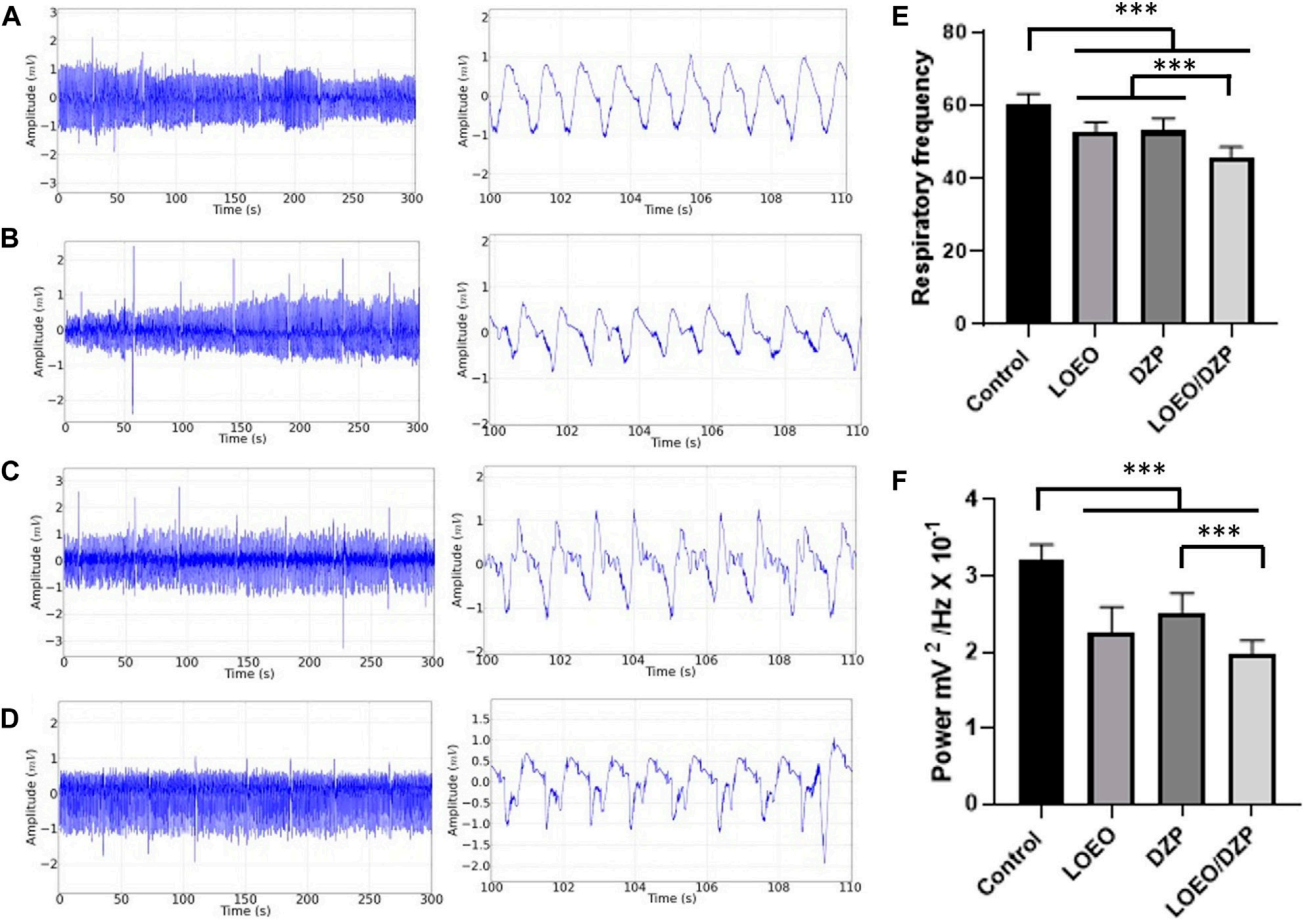
Demonstrations of electromyographic recordings performed in the 10th intercostal muscle of the rat. Control group **(A)**; Lippia origanoide group (LOEO) **(B)**; Diazepam group (DZP) **(C)**; LOEO/DZP group **(D)**; Graph showing the mean respiratory frequency for the groups during treatment **(E)**; Graph depicting the mean power of intercostal muscle contractions in animals subjected to treatment **(F)**. Recordings had a duration of 5 min. After ANOVA and Tukey’s test, (***) *p* < 0.0001.

There was a reduction in respiratory rate when compared to the control group (60.22 ± 2.906/minute), LOEO group (52.44 ± 2.78/minute), DZP group (52.89 ± 3.480/minute) and LOEO/DZP group (45.33 ± 3.162/minute). The LOEO and DZP groups did not show a significant difference (*p* = 0.990), however, there was a decrease in respiratory frequency for the LOEO/DZP combination (Figures 2A–E).

To evaluate the muscle contraction power of the 10th intercostal muscle during treatment, it was observed that the control group had a mean power (3.215 ± 0.196 mV^2^/Hz × 10–1) and presented greater power compared to the other groups: LOEO group (2.254 ± 0.3382 mV^2^/Hz × 10–1), DZP group (2.523 ± 0.2479 mV^2^/Hz × 10–1) and LOEO/DZP group (1.976 ± 0.1767 mV^2^/Hz × 10–1). The LOEO and DZP groups did not show a significant difference (*p* = 0.1181). The LOEO and LOEO/DZP groups were also similar (*p* = 0.1023). The muscle contraction power of the DZP group was greater than that of the LOEO/DZP group (Figure 2F).

### 3.2 Behavioral assessment

The behavioral assessment of the animals was conducted to determine the progression of seizures (Table 1). Animals treated with PTZ rapidly progressed to tonic-clonic seizures with loss of postural reflex.

**TABLE 1:** Behavioral Characterization for Latencies of Excitability Behaviors Induced by PTZ (control group), Diazepam followed by PTZ application, and LOEO followed by PTZ application. (*) indicates statistical difference for the PTZ group, (#) represents statistical difference for the DZP + PTZ group, and (+) represents statistical difference for the LOEO group. After ANOVA followed by Tukey’s test, a significance level of *p < 0.05, **p < 0.001, and ***p < 0.0001 was adopted.

Comportamento/Latência (S)	Whisker piloerection	Orofaciais movements	Generalized tremor	Anterior limb espasms	Isolated clonic seizure without loss of postural reflex	Generalizes clonic seizure with transiente loss of postural reflex	Tonic-clonic seizure with loss of postural reflex
PTZ	59.0 ± 5.809	74.11 ± 8.738	84.67 ± 6.164	96.33 ± 4.637	113.1 ± 8.860	161.1 ± 22.74	207.43 ± 16.32
DZP + PTZ	131.6 ± 19.17***	180.9 ± 10.88***	242.6 ± 25.49***	304.2 ± 40.17***	—	—	—
LOEO + PTZ	117.7 ± 7.14***	153.0 ± 12.96***/###	193.4 ± 12.07***/###	280.3 ± 10.65***	338.7 ± 34.7***	—	—
LOEO/DZP + PTZ	172.0 ± 16.47***/###/+++	—	296.3 ± 21.17***/###/+++	—	—	—	—
*F*-value and *p*-value	*F* (3,34) = 108.9 *p* < 0.0001	*F* (2,24) = 228.3 *p* < 0.0001	*F* (2,24) = 228.2 *p* < 0.0001	*F* (2,24) = 199.8 *p* < 0.0001	—	—	—

The group treated with DZP + PTZ exhibited the longest latency to the onset of convulsive seizures compared to the LOEO + PTZ group. However, when compared to the LOEO/DZP + PTZ combination, it showed significantly shorter latency. In the LOEO + PTZ group, animals did not progress to generalized clonic seizures. In the LOEO/DZP + PTZ group, animals only displayed whisker piloerection and generalized tremor, indicating greater stabilization of convulsive symptoms compared to DZP + PTZ. These results suggest that LOEO, when combined with DZP, can provide effective control of convulsive seizures by potentiating its effects.

### 3.3 Electrocorticographic evaluation

**FIGURE 3:**
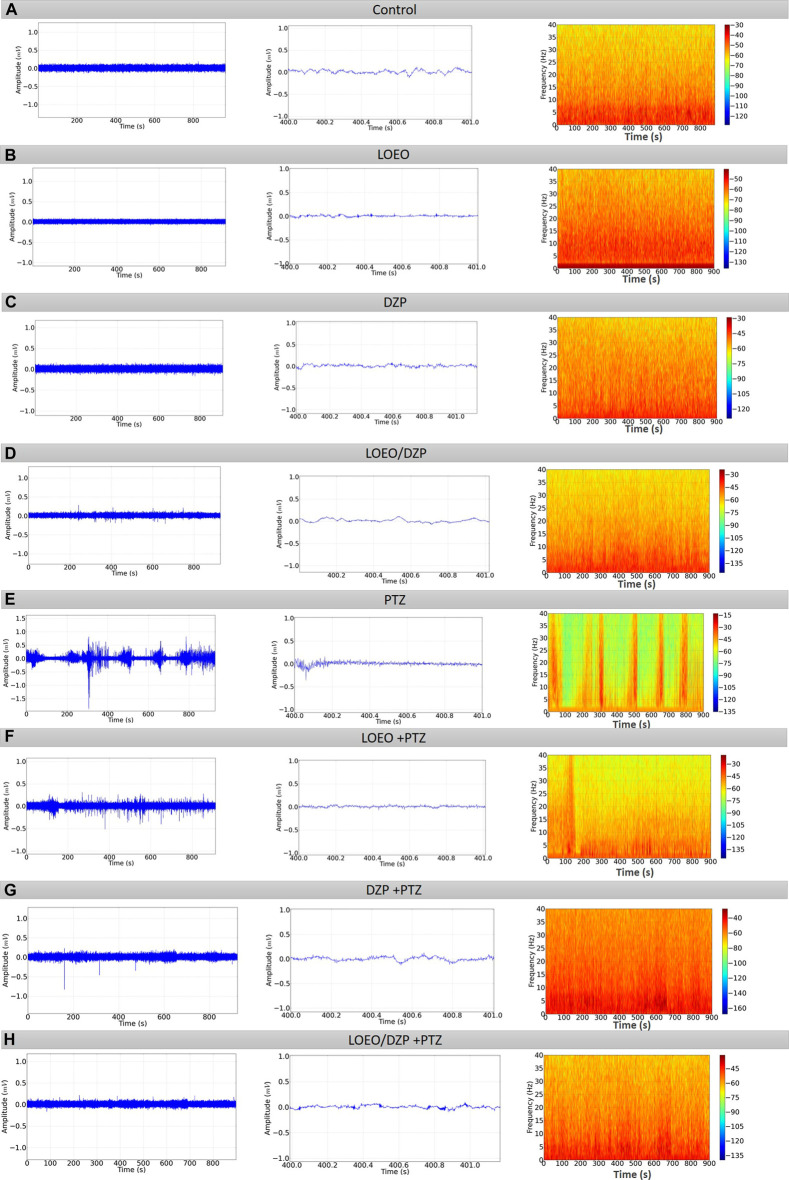
(Continued)

The animals in group I (physiological saline) exhibited amplitudes below 0.1 mV in the trace, and it can be observed in the corresponding spectrogram that the highest energy concentrations are below 10 Hz (Figure 3A). Group II (LOEO) showed little variation compared to the control group, although the spectrogram displayed greater intensity in oscillations up to 40 Hz (Figure 3B). Group III (DZP) displayed oscillations with amplitudes below 0.5 mV in the trace, with energy concentration below 10 Hz (Figure 3C). These groups did not maintain statistical differences and showed trace characteristics similar to the control group. In contrast, group IV (LOEO/DZP) (Figure 3D) displayed oscillations with amplitudes below 0.5 mV in the trace, and energy concentration below 15 Hz. On the other hand, group V (PTZ) exhibited significant alterations in the EEG trace, with peak amplitudes exceeding 0.5 mV, and activities characterized by constant levels of high-frequency and high-amplitude wave peaks (Figure 3E).

For group VI (LOEO + PTZ), the electrocorticographic trace did not show variations above 0.5 mV in amplitude, indicating seizure control. The spectrogram demonstrated an increase in power below 20 Hz (Figure 3F). For group VII (DZP + PTZ), the electrocorticographic trace did not show variations above 0.1 mV in amplitude, indicating seizure control. However, the spectrogram displayed an increase in power below 20 Hz (Figure 3G).

In group VIII, (LOEO/DZP + PTZ), the electrocorticographic trace did not show variations above 0.1 mV in amplitude, possibly indicating potentiation of the effect of DZP when combined with LOEO. The spectrogram displayed an increase in power below 15 Hz (Figure 3H).

**FIGURE 4:**
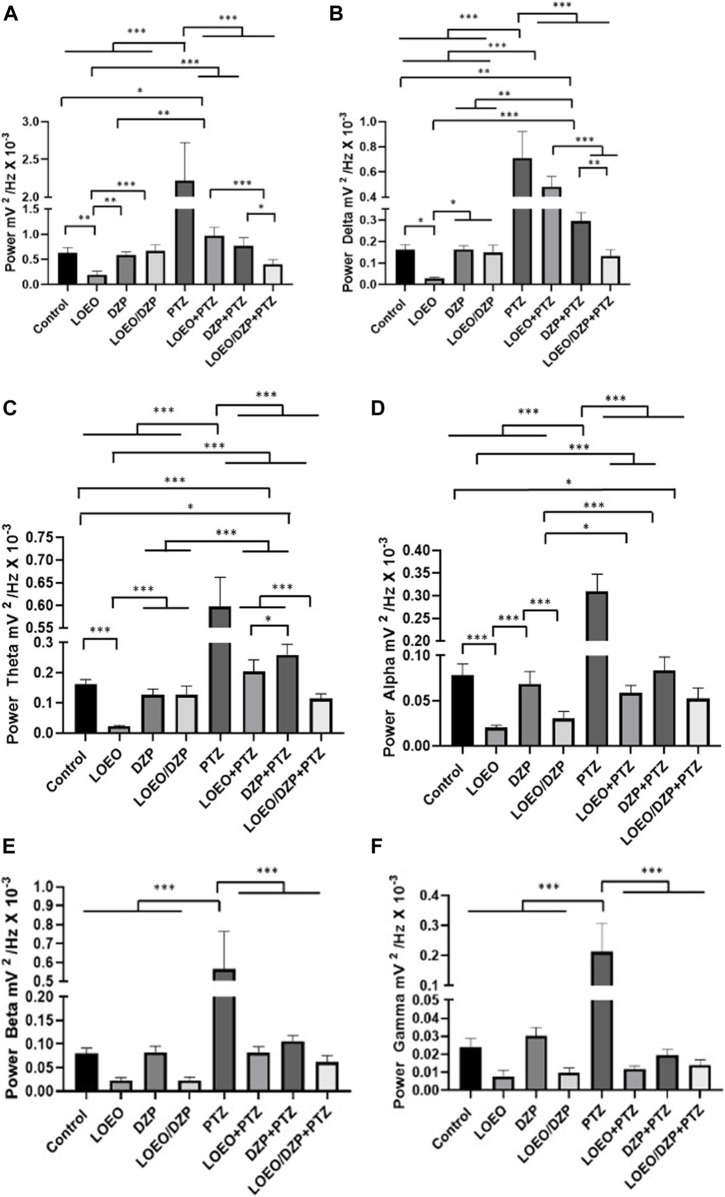
Total linear power analysis of brain waves up to 40 Hz **(A)** and quantitative linear frequency distribution of brain waves: **(B)** delta waves; **(C)** theta waves; **(D)** alpha waves; **(E)** beta waves and **(F)** gamma waves, recorded by electrocorticography. Data show drugs associated and not associated with pentylenetetrazole (*p < 0.05, **p < 0.001 and ***p < 0.0001).

Total power varied significantly between groups: group I 0.6268 ± 0.1064 mV^2^/Hz × 10⁻³ and group II 0.1946 ± 0.06929 mV^2^/Hz × 10⁻³ (*p* = 0.0012), group VI 0.9680 ± 0.1696 mV^2^/Hz × 10⁻³ (*p* = 0.0211). However, groups III 0.5868 ± 0.06176 mV^2^/Hz × 10⁻³ (*p* > 0.9999), group IV 0.6718 ± 0.1204 mV^2^/Hz × 10⁻³ (*p* = 0.9998), group VII 0.7690 ± 0.1624 mV^2^/Hz × 10⁻³ (*p* = 0.8363), and group VIII 0.4031 ± 0.09328 mV^2^/Hz × 10⁻³ (*p* = 0.3313) no showed a difference to the saline control group. The administration of PTZ 2.215 ± 0.5042 mV^2^/Hz × 10⁻³ (group V) resulted in a significant increase in power and presented a statistical difference in relation to all groups [F (7,64) = 77.25; *p* < 0.001] (Figure 4A).

Significant variation was found between groups II and VI (LOEO and LOEO + PTZ), groups II and VII (LOEO/DZP + PTZ), and between group VI and VIII (LOEO + PTZ and LOEO/DZP + PTZ).

For delta oscillations, the control group presented an average power of 0.1609 ± 0.02492 mV^2^/Hz × 10⁻³, showing significant variation between the following groups: group II with 0.02884 ± 0.004213 mV^2^/Hz × 10⁻³ (*p* = 0.0276) and group VII with 0.2948 ± 0.03959 mV^2^/Hz × 10⁻³ (*p* = 0.0241). However, it was similar to groups III with 0.1624 ± 0.01824 mV^2^/Hz × 10⁻³ (*p* > 0.9999), group IV with 0.1487 ± 0.03489 mV^2^/Hz × 10⁻³ (*p* > 0.999) and group VIII with 0.1313 ± 0.03135 mV^2^/Hz × 10⁻³ (*p* = 0.9949). However, significant differences were observed between groups V with 0.7095 ± 0.2105 mV^2^/Hz × 10⁻³ and VI with 0.4827 ± 0.08197 mV^2^/Hz × 10⁻³, for the other groups (Figure 4B).

For theta oscillations, group I presented an average power of 0.1626 ± 0.01493 mV^2^/Hz × 10⁻³ and showed no statistical difference with group III 0.1272 ± 0.01868 mV^2^/Hz × 10⁻³ (*p* = 0.3057), group IV 0.1275 ± 0.02840 mV^2^/Hz × 10⁻³ (*p* = 0.3139) and group VI 0.2043 ± 0.03789 mV^2^/Hz × 10⁻³ (*p* = 0,1322). Significant statistical differences were observed between groups II 0.1626 ± 0.01493 mV^2^/Hz × 10⁻³, V 0.5975 ± 0.06429 mV^2^/Hz × 10⁻³ and VII 0.2595 ± 0.03483 mV^2^/Hz × 10⁻³ and group VIII 0.1142 ± 0.01620 mV^2^/Hz × 10⁻³ (*p* = 0.0470) (Figure 4C).

For alpha oscillations, group I presented an average linear power of 0.07834 ± 0.01241 mV^2^/Hz × 10⁻³ and showed no statistical difference with group III (0.06866 ± 0.01372 mV^2^/Hz × 10⁻³, *p* = 0.9226), group VI (0.05895 ± 0.008050 mV^2^/Hz × 10⁻³, *p* = 0.2384) and group VII (0.08349 ± 0.01473 mV^2^/Hz × 10⁻³, *p* = 0.9980). Significant statistical differences were observed between groups II (0.02046 ± 0.002646 mV^2^/Hz × 10⁻³), group IV (0.03024 ± 0.008092 mV^2^/Hz × 10⁻³) and group V (0.3096 ± 0.03782 mV^2^/Hz × 10⁻³) and group VIII (0.05244 ± 0.003926 mV^2^/Hz × 10⁻³). The PTZ group had a higher mean linear power in alpha oscillations (Figure 4D).

For beta oscillations, the control group exhibited an average linear power of 0.08061 ± 0.01036 mV^2^/Hz × 10⁻³, with no statistical difference observed in comparison to group III (0.08187 ± 0.01351 mV^2^/Hz × 10⁻³, *p* > 0.9999), group IV (0.02291 ± 0.007150 mV^2^/Hz × 10⁻³, *p* = 0.6753), group VI (0.08120 ± 0.01311 mV^2^/Hz × 10⁻³, *p* > 0.9999), group VII (0.1060 ± 0.01166 mV^2^/Hz × 10⁻³, *p* = 0.9948), and group VIII (0.06128 ± 0.01399 mV^2^/Hz × 10⁻³, *p* = 0.9991). Significant statistical difference was observed only in group V (0.5655 ± 0.1993 mV^2^/Hz × 10⁻³) (Figure 4E).

For gamma oscillations, the control group exhibited an average linear power of 0.02402 ± 0.004807 mV^2^/Hz × 10⁻³, with no statistical difference observed for group II (0.007608 ± 0.003549 mV^2^/Hz × 10⁻³, *p* = 0.9667), group III (0.03010 ± 0.004594 mV^2^/Hz × 10⁻³, *p* > 0.9999), group IV (0.009768 ± 0.002647 mV^2^/Hz × 10⁻³, *p* = 0.9849), group VI (0.01174 ± 0.001755 mV^2^/Hz × 10⁻³, *p* = 0.9938), group VII (0.01953 ± 0.003204 mV^2^/Hz × 10⁻³, *p* > 0.9999), and group VIII (0.01391 ± 0.002762 mV^2^/Hz × 10⁻³, *p* = 0.9982). Significant statistical difference was observed only in group V (0.2121 ± 0.09447 mV^2^/Hz × 10⁻³) (Figure 4F).

## 4 Discussion

In this study, we have demonstrated, for the first time, that the essential oil of Lippia origanoides was able to increase the convulsive threshold induced by PTZ in rats. This was achieved through the analysis of behavior, electroencephalographic (EEG) and electromyographic patterns after the administration of LOEO alone, as well as the evaluation of the LOEO/DZP combination and its response compared to diazepam.

The present study demonstrated significant differences in the respiratory frequency depression of the group treated with LOEO in combination with DZP compared to the other groups in relation to respiratory behavior patterns, as assessed through electromyography of the 10th intercostal muscle.

These respiratory depressant effects caused by the LOEO/DZP combination suggest various therapeutic targets. Some anesthetic medications are known respiratory depressants, such as propofol, sevoflurane, and midazolam, acting as GABA receptor agonists and NMDA receptor antagonists (Pattinson, 2008).

The depressant respiratory response shown in our results by the LOEO/DZP combination suggests the need for further investigations to elucidate the underlying mechanisms triggering the decrease in respiratory frequency after its combined administration.

In recent a study (Hamoy et al., 2018), behavioral and electrocorticographic assessments were extremely useful to evaluate and compare the changes arising from disordered neuronal excitability that generate seizures and consequent epileptic conditions. In this research, the electrocorticogram of rat cortex was examined and it was demonstrated that the administration of PTZ in rats presented continuous discharges and high amplitude waves, being this effect reversed by conventional anticonvulsants (phenobarbital, phenytoin and diazepam). Our results corroborate with the whole protocol of this study, using diazepam as conventional antiepileptic.

Thymol (2-isopropyl-5-methylphenol), the most abundant constituent (57.46%) in LOEO and several other essential oils, is an isomer of carvacrol (2-methyl-5-1 methylethylphenol), a monoterpene also present in LOEO (1.42%). Thymol can manifest as a white crystalline powder or colorless crystals and are constituents of the essential oils of several plants (Nunes et al., 2005; Guillen et al., 2007).

The effects of thymol that have been studied and described in the literature include its antimicrobial and antiseptic actions (Matos et al., 2000; Kachur and Suntres, 2020). Both carvacrol and thymol exhibit high antioxidant activity, serving as natural food preservatives that inhibit peroxidation of phospholipid liposomes and demonstrating antifungal activities (Milos et al., 2000; Teissedre and Waterhouse, 2000; Mastelic et al., 2008). Other natural monoterpenoids have a wide range of pharmacological properties, such as local anesthetic, anticancer, antihistaminic, anti-inflammatory, antiviral, and neuroprotective activities (Volcho et al., 2018).

In previous studies on the central action of carvacrol, its effects on experimental models of anxiety and depression in mice were demonstrated, suggesting involvement of the GABAergic system through the GABA-A receptor, similarly to benzodiazepines that have high affinity for these receptors. In evaluating the antidepressant effects of carvacrol, the mechanism of action was shown to be associated with the dopaminergic system, possibly through stimulation of D1 and D2 receptors (Melo et al., 2010; Melo et al., 2011). Other studies have also shown central nervous system actions of monoterpenes, exhibiting anxiolytic and antidepressant effects (Umezu and Morita, 2003; Silva et al., 2007).

In a study the authors suggested that the mechanism of action of the isomers carvacrol and thymol is related to the modulation of GABAergic ionotropic receptors with Cl^−^ channels, as the monoterpenes bound to GABA receptors increased the uptake of ^36^Cl^−^ (Tong and Coats, 2010).

Analogues of carvone, such as carvacrol, were able to inhibit neuronal excitability in the sciatic nerve of rats, probably by blocking voltage-dependent sodium channels. The authors also observed that the structure of the compounds interfered with their ability to block channels. This capability of these compounds to alter their chemical structures can be effective in delivering drugs that act directly on their targets, without affecting other organisms (Gonçalves et al., 2010).

To evaluate the mechanisms by which carvacrol promoted the inhibition of neuronal excitability, the authors demonstrated through several tests that carvacrol is able to block neuronal excitability in a reversible and concentration-dependent manner through direct inhibition of voltage-dependent sodium channels, which suggests its effect as a local anesthetic (Joca et al., 2012).

Studies using oils and plant extracts have demonstrated the potentiation of GABAergic pathways in the control of convulsive crises triggered by pentylenetetrazole (de Oliveira et al., 2022; Muto et al., 2022; Nascimento et al., 2022). This effect is allosterically potentiated by benzodiazepines such as Diazepam, which favors the hyperpolarization of the neuronal membrane (Aburawi et al., 2021). LOEO demonstrated the ability to mitigate the intensity of PTZ-induced seizures, increasing the latency for the onset of behavior, as evidenced by ECoG. It was observed that the anticonvulsant activity of LOEO increased seizure control when associated with Diazepam, which demonstrated a potentiating effect on seizure control.

In summary, the outcomes of this study underscore the potential utility of LOEO in managing convulsive seizures, while its synergistic combination with DZP opens a promising pathway for the development of new agents targeting refractory epilepsy. Moreover, these findings contribute significantly to the deeper comprehension of the mechanisms underlying epilepsy.

## 5 Conclusion

The current study revealed that treatment with LOEO led to distinct alterations in electrocorticographic tracings, showcasing attributes of a potent anticonvulsant agent. Moreover, the combination of LOEO with diazepam yielded a more favorable response compared to any individual drug administration, resulting in increased convulsive threshold and respiratory depression. This finding holds significant implications, as the synergistic effect of Lippia origanoides essential oil with diazepam may represent a valuable therapeutic approach for the treatment of epilepsy, enhancing therapeutic efficacy while minimizing adverse effects.

## Data Availability

The original contributions presented in the study are included in the article/supplementary material, further inquiries can be directed to the corresponding authors.
